# Functional genomic analysis of *Vreelandella zhaodongensis* BS253: a new isolate from hypersaline lakes from Brazilian Pantanal

**DOI:** 10.1007/s00203-026-05318-2

**Published:** 2026-10-31

**Authors:** William Lautert-Dutra, Amanda Pasinato Napp, Eduardo Alexandre Back Sivinski, Charley Christian Staats, Francine Melise dos Santos, Clarissa Lovato Melo

**Affiliations:** 1 Institute of Petroleum and Natural Resources (IPR), Pontifical Catholic University of Rio Grande do Sul (PUCRS), Porto Alegre, Brazil; 2 Graduate Program in Cellular and Molecular Biology, Center of Biotechnology, Federal University of Rio Grande do Sul, Porto Alegre, Brazil

**Keywords:** Environmental microbiology, Microbial biotechnology, Halomonadaceae, Microbial surfactants, Bioemulsifiers, Sustainable bioprocessing, Microbial genomics, Functional genomics, Halophile

## Abstract

Natural ecosystems harbor an immense diversity of microorganisms whose metabolic capabilities remain unexplored. Therefore, the investigation of microbial life in diverse environments can reveal bacteria with unique physiological and biochemical traits, which may be useful for biotechnological applications. In this study, we report the complete genome sequencing and functional characterization of isolate BS253, isolated in a hypersaline alkaline lake from Brazil’s Pantanal region. Using a hybrid sequencing strategy combining Oxford Nanopore long reads and Illumina short reads, we assembled a circular chromosome of 3.76 Mb and two plasmids. Phylogenetic and comparative genomic analyses identified the isolate as *Vreelandella zhaodongensis*. Digital DNA-DNA hybridization (76.4%) and ANI (96.83%) values supported the designation of BS253 as a strain of *V. zhaodongensis*. Genome mining revealed genes potentially associated with salt and alkali tolerance, hydrocarbon and plastic degradation, and the biosynthesis of secondary metabolites. Phenotypically, BS253 is a moderately halophilic, facultatively anaerobic, Gram-negative rod that also exhibits emulsifying activity (55.1%) under defined culture conditions. These findings highlight strain BS253 as a metabolically versatile extremophile and a promising candidate for future studies in environmental and industrial biotechnology.

**Supplementary Information:**

The online version contains supplementary material available at https://doi.org/10.1007/s00203-026-05318-2.

## Introduction

The interest in microorganisms adapted to extreme environments has become increasingly relevant for both ecological understanding and biotechnological innovation. Extreme environments exert selective pressures on microorganisms, which, in turn evolve unique molecular adaptations that allow them to survive. Among these adaptations are specialized metabolites, a broad category of natural products that contribute to the ability of the organism to adapt to their surrounding environment, and extracellular amphiphilic biopolymers, such as bioemulsifiers (Katz and Baltz 2016; Shu and Huang 2022). Biosurfactants lower the surface and interfacial tension in biphasic systems, facilitating processes like emulsification and dispersion. These molecules have been described in microorganisms from hypersaline lakes and salterns, cold environments, deep-sea hydrothermal vents, petroleum reservoirs, and alkaline habitats (Poli et al. 2010). Because they remain active under salinity, temperature, and pH conditions like those found in industrial settings, bioemulsifiers are considered promising candidates for industrial bioprocesses and bioremediation (Napp et al. 2026b).

The isolation of microbial organisms from extreme environments is pivotal for the discovery of new strains with potential for ecological and biotechnological applications (Finore et al. 2015; Shu and Huang 2022). In Brazil, the Pantanal region represents one of the largest tropical wetland ecosystems in the world and hosts a complex mosaic of aquatic environments, including hypersaline lakes. These habitats are influenced by seasonal flooding, high evaporation rates, and fluctuating physicochemical parameters, which contribute to the establishment of highly adapted microbial communities (Guerreiro et al. 2019). Such environments harbor unique halophilic and alkaliphilic microorganisms with metabolic versatility. However, the microbial biodiversity of hypersaline lakes of the Pantanal remains poorly studied, which prevent full characterization of the valuable reservoir of novel taxa and bioactive molecules (Andreote et al. 2018; Guerreiro et al. 2019; Cotta et al. 2022).

In this regard, halophilic organisms produce a plethora of biotechnologically relevant molecules (Margesin and Schinner 2001). For instance, the family Halomonadaceae, the largest group of halophilic microorganisms, is known to produce a wide range of biotechnologically relevant molecules, including lipids, enzymes, and polysaccharides, that are applicable in various industrial processes (Ma et al. 2020; Biswas et al. 2023). Within this family, the *Vreelandella* genus was recently proposed as one of five new genera from *Halomonas*, currently comprises 41 species validly published under the International Code of Nomenclature of Prokaryotes, together with four species whose names have not been validly published, totaling 45 species (Göker et al. 2025). The species show Gram-negative rod cells, aerobic or facultatively anaerobic metabolism, slight to moderately halophilic, alkaliphilic or alkali-tolerant, growing at pH values in the range of 5.0–12.0 (de la Haba et al. 2023). For example, recent work with *V. zhaodongensis* BS253 showed that inorganic carbon availability modulates extracellular emulsification activity and the expression of genes annotated as related to exopolysaccharide biosynthesis (Napp et al. 2026a).

To expand the genomic and functional knowledge of halophilic bacteria from the Brazilian Pantanal, we investigated strain BS253, an emulsifier-producing isolate recovered from a hypersaline alkaline lake. This study characterized the complete genome of BS253 using a hybrid sequencing strategy combining Oxford Nanopore long reads and Illumina short reads. We determined its phylogenomic placement within the genus *Vreelandella*, and integrated phenotypic, biochemical, and functional genomic analyses to uncover its ecological adaptations and biotechnological potential of this organism, including its possible future relevance to industrial bioprocesses.

## Materials and methods

### Bacterial isolation and growth conditions

**Fig. 1:**
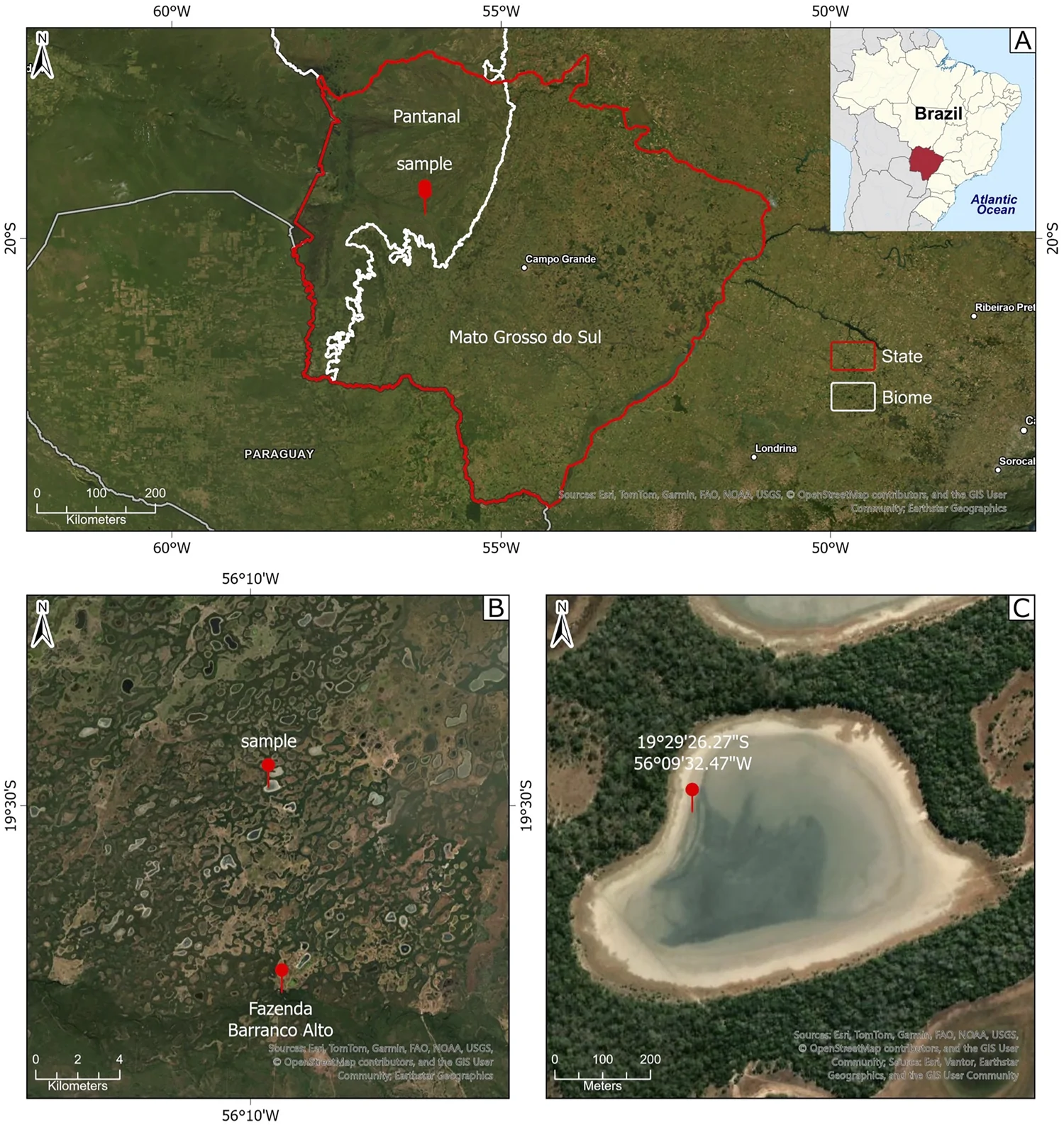
Geographic location of the sampling site in the Brazilian Pantanal. **A** Regional overview showing the state of Mato Grosso do Sul (red outline) and the Pantanal biome (white outline) in central-western Brazil; the inset locates the region within Brazil. **B** Detailed view of the sampling area within the *Pantanal Nhecolândia* region, indicating the sampling point and the nearby reference locality of *Fazenda Barranco Alto*. **C** High-resolution view of the hypersaline alkaline lake from which strain BS253 was isolated, with the exact collection point marked (19°29′26.27″ S, 56°09′32.47″ W). Sediment was collected from the upper layer of a push-core sample during the Pantanal “*vazante*” period (June 2017), when lake water showed a salinity of 4,710 ppm and pH 10.93. North arrows and scale bars are shown in each panel

Strain BS253 was isolated from the upper layer of a sediment core collected from a hypersaline alkaline lake in the Pantanal Nhecolândia region, Brazil (19°29′26.27″ S, 56°09′32.47″ W), using push-core sampling. Sediment samples were collected in June 2017, during the Pantanal “*vazante*” period, which marks the transition from the flooding to the dry season (Fig. 1). At the time of sampling, lake water showed a salinity of 4,710 ppm and a pH of 10.93, measured in the field with a QED MP20 multiparameter instrument. The cuvette and sensors were rinsed with deionized water between measurements. Isolation was performed using the top layer of the sediment core inoculated into saline medium composed of 10 g/L yeast extract, 100 g/L NaCl, 3 g/L sodium citrate, 2 g/L KCl, 1 g/L MgSO_4_, 280 µg/L MnCl_2_, 0.05 g/L FeSO_4_, and 3 g/L NaHCO_3_ (Poli et al. 2007). Cultures were incubated at 28 °C with agitation at 80 rpm until turbidity was observed, approximately after 3 days. Serial dilutions were then performed, and the diluted cultures were streaked onto solid saline medium and incubated at 28 °C to obtain isolated colonies. Culture purity was assessed based on colony morphology and cellular morphology by Gram staining, as described below. Cultures showing distinct colony morphologies or cellular morphotypes were subjected to successive streaking until morphologically homogeneous cultures were obtained. Among the recovered isolates, strain BS253 was selected for further characterization based on a preliminary screening that considered consistent growth, culture purity, reproducibility, and emulsifying activity.

### Microbial phenotypic and biochemical features

The physiological and biochemical properties of strain BS253 were characterized following standard microbiological protocol (Arahal et al. 2007). For Gram staining, cells cultivated in modified LB medium (1.0% tryptone and 0.5% yeast extract, supplemented with 1%, 5%, or 10% NaCl) were stained using the Gram-Color modified staining kit (Merck, Darmstadt, Germany), according to the manufacturer’s instructions. Cellular morphology was assessed from stained preparations by bright-field light microscopy using a Nikon Eclipse Si microscope and cell dimensions were measured from 50 individual cells using ImageJ software. Colony morphology was evaluated on modified LB agar plates under the same NaCl concentrations. Motility was assessed using API M Medium (bioMérieux), according to the manufacturer’s instructions. Conventional phenotypic characterization included oxidase and catalase activities, hydrolysis of starch, Tween 80, and urea, and growth under different NaCl, pH, and temperature conditions, following the procedures described by Mata et al. (2002). Anaerobic growth was evaluated by transferring actively growing colonies of strain BS253 to fresh solid saline medium, followed by incubation at 28 °C for 21 days in an anaerobic jar (Permution^®^, model JA0403) containing a GENbox Anaer^®^ gas-generating system (bioMérieux). Growth under anaerobic conditions was assessed based on visible colony development. Biochemical profiling was further complemented using the API 20E and API 20NE miniaturized systems (bioMérieux), according to the manufacturer’s instructions.

The growth optimization was evaluated in a modified LB medium supplemented with different NaCl concentrations (0, 3, 4, 5, 10, 12.5, 15 and 20%). To determine the pH range compatible with growth, the culture medium was adjusted to pH values from 3 to 13 at one-unit intervals using specific buffering systems. The thermal tolerance range was evaluated by incubating the cultures at 10, 20, 30, 40, and 50 °C (Jiang et al. 2013). Antibiotic susceptibility was evaluated as a qualitative assay under antibiotic exposure. Briefly, strain BS253 was grown in LB medium and standardized to an initial OD_600_ of 0.05. Cultures were incubated in LB medium supplemented with ampicillin, kanamycin, chloramphenicol, or tetracycline at final concentrations of 50 and 100 µg/mL. During incubation at 28 °C and 180 rpm, aliquots were collected at 24, 48, and 72 h and plated onto LB agar containing the corresponding antibiotic concentration and growth was evaluated qualitatively by colony formation.

### Genomic DNA preparation and sequencing

For long-read sequencing, isolated colonies were collected and suspended in 1mL of Tris-EDTA buffer solution (Sigma-Aldrich; St. Louis, Missouri, USA) and sent to the Life Sciences Core Facility (LaCTAD; Campinas, Brazil) for DNA extraction, library preparation, and whole-genome sequencing. Briefly, QIAcube^®^ (QIAGEN, QIAamp DNA Mini Kit) equipment was used for DNA isolation according to the manufacturer’s instructions. Then, DNA quality was evaluated using the Agilent TapeStation system (Agilent Technologies; Santa Clara, CA, United States). Extracted DNA showed a DNA Integrity Number (DIN) of 8.1 and a concentration of 22.0 ng/µL, with most of the sample (87% of the integrated area) corresponding to high-molecular-weight fragments (~ 52 kb). Whole-genome sequencing was performed using Nanopore Long Read technology (Oxford Nanopore Technologies; Oxford, UK). A Nanopore library was constructed using Native Barcoding Kit 96 V14 (SQK-NBD114.96). Library sequencing was done using FLO-PRO114M: PromethION R10 (M Version).

For short-read sequencing, the isolated colonies were sent to Neoprospecta Microbiome Technologies for DNA extraction using NeoSampleX^®^ lysis buffer and DNA extraction with magnetic beads. Sequencing was done using Illumina NextSeq 1000 (2 × 150 bp, P1-600 Illumina kit).

### Genome and plasmid assembly

The quality and filtering of PE raw reads were analyzed using *FastQC* v0.11.9 and *fastp* v0.23.4 (*trim-poly-g -detect-adapter minlength 90 bp*) (Andrews 2010; Chen et al. 2018). Long-reads were filtered by quality using *Filtlong* v0.2.1 (Wick and Menzel 2017), using the short reads as a reference for *k*-mer matching (‘*PE short-reads’* + *--min_length 1000 --keep_percent 90*) to keep only high-quality long reads. Filtered nanopore reads were assembled using *Trycycler* v0.5.5 (Wick et al. 2021), which relies on multiple independent long-read assemblies of the same genome and produces a final consensus *de novo* long-read genome assembly. The *Flye* v2.9.5-b1801 (Kolmogorov et al. 2019), *Minipolish* (*miniasm_and_minipolish.sh*) v0.1.2 (Wick and Holt 2021), and *Raven* v1.8.3 (Vaser and Šikić 2021) assemblies were chosen for multi-assembly. Assemblies were manually curated using *Bandage* v0.8.1 (Wick et al. 2015) and fragmented genomes were discarded. After the sequential steps, the final consensus was polished using *Medaka* v2.0.1 and a short-read polishing using *Polypolish* v0.6.0 and *Pypolca* v0.3.1 (Bouras and Wick 2024; Bouras et al. 2024). The presence of plasmids was evaluated using *Plassembler* v1.6.2 (Bouras et al. 2023). This method uses a hybrid approach to remove chromosomal reads and automatically assemble plasmids. Assembled plasmids were annotated following the same approach of the genome sequence (Table S1).

### Genome annotation

Genome sequencing annotation was done using *PGAP* v*2024-07-18.build7555* (NCBI Prokaryotic Genome Annotation Pipeline; third-party software used by the tool: *tRNAscan-SE*,* hmmer*,* CRISPCasFinder*,* AntiFam*,* Rfam*,* GeneMarkS2*,* infernal*,* CheckM*: HMMs libraries: TIGRFAM, Pfam, PRK HMMs) (Li et al. 2021). To assess the genome completeness and contamination, we used the Benchmarking Universal Single-Copy Orthologue (*BUSCO*) tool v5.8.2 and *CheckM v1.2.5* to provide completeness and redundancy analysis related to expected gene content in the *de novo* assembled genome and annotated gene set (Parks et al. 2015; Simão et al. 2015). Assembly quality and coverage were evaluated using *QUAST* v5.2.0 (Gurevich et al. 2013). We used *antiSMASH* v7 to annotate secondary metabolite biosynthesis gene clusters (Blin et al. 2023). The presence of Toxin-antitoxin pairs was annotated using *TAfinder* 2.0 (Xie et al. 2018). Finally, we investigated the antibiotic resistance genes presented in the BS253 genome using the Comprehensive Antibiotic Resistance Database (CARD) (Alcock et al. 2023).

### Phylogenetic and genome similarity analysis

Phylogenomic analysis was used to assess the evolutionary relationship among species of the *Vreelandella* genus. First, after-tax identification by *PGAP* (-*-taxcheck*) in the annotation step, we downloaded RefSeq reference genomes from the assigned genes (*Vreelandella*) (Additional file: Table S2) using the *datasets* v16.40.1 tool (O’Leary et al. 2024). Then, the BS253 and reference genomes were mapped to the *Phylophlan* database (Asnicar et al. 2020) using *DIAMOND* v2.1.11.165 (Buchfink et al. 2021). Multi-fasta mapped files for core genes were aligned and trimmed using *MUSCLE* (v5.3.linux64) and *trimAL* (v1.5.rev0 build [2024-05-27]) (Edgar 2004; Capella-Gutiérrez et al. 2009). These files were used to compute independent gene trees using *RaxML* v8.2.12 (ML + LG matrix-based model) for all genes (Stamatakis 2014). The final trees were trimmed using *TreeShrink* v1.3.9 with different false positive error rates (*-q* “0.05, 0.10, 0.10”) options. After, each final evolutionary species tree was generated using *ASTRAL* v5.7.8 (Zhang et al. 2018). The ASTRAL normalized score was used to choose the best species tree. The *Carnimonas nigrificans* (Accession: GCF_000526695.1) genome was used as an outgroup (Arahal et al. 2007).

To further determine the relationship of strain BS253 to the previously described species and following the minimal standards for prokaryote taxonomy (Chun et al. 2018), Average Nucleotide Identity (ANI) was calculated for the BS253 and reference sequences (Table S2) with *fastANI* v1.34 (Jain et al. 2018). Additionally, the BS253 genome sequence was uploaded to the TYGS web server for dDDH (digital DNA-DNA hybridization) computed with GGDC (Genome-to-Genome Distance Calculator), using the average dDDH from _*d*_0, _*d*_4, and _*d*_6 (Meier-Kolthoff et al. 2013; Meier-Kolthoff and Göker 2019). 16 S rRNA comparison were performed using the EzBioCloud.

### Microbial secondary metabolites and special metabolism analysis

The presence of specialized metabolism linked to hydrocarbon degradation, plastic degradation, and biosurfactant production was determined using *HADEG* (*HADEG_protein_database_231119.faa*) (Rojas-Vargas et al. 2023). Also, we characterized the presence of Toxin-antitoxin (TA) loci presented in the genome using the TADB database web server (Guan et al. 2024).

Microbial surfactant production was evaluated using experimental methods. First, the strain BS253 was initially cultured in Saline medium (Poli et al. 2007) for 24 h. Next, the cells were harvested by centrifugation at 10,000 rpm for 10 min and washed twice with sterile saline solution (0.9% NaCl). The washed cells were then resuspended in MSM medium (Brito et al. 2024) supplemented with 2% glucose as a carbon source. The cell suspension [1% (v/v)] was used as the inoculum for microbial surfactant induction. The experiment was incubated for 5 days at 28 °C and 180 rpm. The ability to produce microbial surfactants was evaluated using the emulsification activity, drop collapse, and oil spreading assays (Cooper and Goldenberg 1987; Youssef et al. 2004; Ben Belgacem et al. 2015). Sterile culture medium and 0.5% SDS were used as the negative and positive controls, respectively. All experiments were performed in biological triplicate. The emulsification index (E24) values were statistically analyzed using GraphPad Prism software.

### Comparative functional genomics

The functional repertoire of strain BS253 was compared with that of its four closest relatives, identified in the phylogenomic and ANI analyses (Fig. 3; Additional file: Table S2): *V. zhaodongensis* 9-2T (GCF_013415115.1), *V. alkaliphila* (GCF_033842765.1), *V. venusta* (GCF_015689495.1) and *V. andesensis* (GCF_003989795.1). RefSeq protein and GFF3 annotation files for the five genomes were retrieved from the NCBI FTP server. Orthologous groups were inferred from the five proteomes with OrthoFinder v3.1.5 using DIAMOND v2.2.8 for the all-versus-all similarity search. Gene families were classified as core (present in all five genomes), accessory (present in two to four genomes), or strain-specific (present in a single genome, including OrthoFinder singletons). COG functional categories were assigned to every predicted protein with COGclassifier v2.0.0, which performs an RPS-BLAST search against the COG position-specific scoring matrices of the NCBI Conserved Domain Database (e-value ≤ 0.01); category frequencies are expressed as the percentage of COG-classified proteins of each genome. Strain-specific proteins of BS253 were mapped back onto the three replicons and grouped into genomic islands using a maximum intergenic distance of 20 kb.

The hydrocarbon-, aromatic-, and plastic-degradation genes reported in Table 3 were in the BS253 proteome by reciprocal BLASTp searches against the HADEG protein database (HADEG_protein_database_231119.faa). The BS253 proteome was first searched against HADEG (e-value ≤ 1 × 10^− 10^, ≥ 30% identity) and the HADEG reference proteins were then searched against the BS253 proteome (e-value ≤ 1 × 10^− 5^, ≥ 25% identity) to recover divergent copies; both searches required at least 50% coverage of query and subject. Orthologues of the resulting BS253 proteins in the four reference proteomes were then defined as reciprocal best BLASTp hits (e-value ≤ 1 × 10^− 5^; ≥ 50% coverage of query and subject; no identity floor applied), and the amino-acid identity and query coverage of each pair were recorded. HADEG reference proteins without a detectable protein-level match were additionally searched against the complete BS253 genome by tBLASTn (e-value ≤ 1 × 10^− 3^) to exclude annotation artefacts. All scripts and result tables are available with the supplementary material.

## Results

### Phenotypic and biochemical characterization of strain BS253

Cells of strain BS253 were identified as Gram-negative, facultative anaerobic, and non-motile rods. Colony morphology varied slightly with salt concentration but was consistently described as cream or beige-colored, circular, convex, exhibiting an entire margin, smooth surface, opaque consistency, and a dull appearance. Colony diameters varied according to NaCl concentration in the modified LB medium: 2.3–3.5 mm at 1% NaCl, 1.8–2.8 mm at 5% NaCl, and 0.6–1.2 mm at 10% NaCl. Cell size was also influenced by salinity, with rod-shaped cells measuring 1.2–2.0 × 0.5–0.8 μm in 1% NaCl, 1.3–2.4 × 0.6–0.9 μm in 5% NaCl, and 1.0–1.6 × 0.5–0.7 μm in 10% NaCl.

**Table 1:** Genome statistics

Step	Features	Value	GCF_013415115.1
Assembly (Chr.)	Genome size (bp)	3,765,793	3.7 Mb
	DNA G + C (%)	53.35	53
	DNA scaffolds	1	25
	N50	3,765,793	352.3 kb
	N90	3,765,793	-
	L50	1	4
	L90	1	-
	N’s	0	-
	Avg. Coverage (Illumina)	529x	200x
	Avg. Coverage (Nanopore)	267x	-
Assembly (Plasmid)	Plasmid 1 length (bp)	28,749	-
	Plasmid 1 Coverage (Illumina)	575x	-
	Plasmid 1 Coverage (Nanopore)	267x	-
	Plasmid 1 Copy Number	1.09	-
	Plasmid 2 length (bp)	6228	-
	Plasmid 2 Coverage (Illumina)	496x	-
	Plasmid 2 Coverage (Nanopore)	173x	-
	Plasmid 2 Copy Number	0.94	-
PGAP identification		*Vreelandella zhaodongensis* (ANI = 96.83%)	*V. zhaodongensis* (ANI = 99.96%)
Genome Annotation (PGAP)	Genes (total)	3,417	3,359
	CDSs (total)	3,335	3,295
	Genes (coding)	3,288	3,289
	CDSs (with protein)	3,288	3,289
	Genes (RNA)	82	64
	rRNAs	6, 6, 6 (5 S, 16 S, 23 S)	2, 1, 1
	Complete rRNAs	6, 6, 6 (5 S, 16 S, 23 S)	1, 1, 1
	tRNAs	60	56
	ncRNAs	4	4
	Pseudo Genes (total)	47	5
	CDSs (without protein)	47	6
	Pseudo Genes (ambiguous residues)	0 of 47	0 of 6
	Pseudo Genes (frameshifted)	16 of 47	2 of 6
	Pseudo Genes (incomplete)	35 of 47	5 of 6
	Pseudo Genes (internal stop)	1 out of 47	0 of 6
	Pseudo Genes (multiple problems)	5 of 47	1 of 6
	CRISPR Arrays	1	1

The summary highlights the major statistics for each step from *de novo* genome assembly, plasmid assembly, to genome annotation. Third-party software used by PGAP: *tRNAscan-SE*,* hmmer*,* CRISPCasFinder*,* AntiFam*,* Rfam*,* GeneMarkS2*,* infernal*,* CheckM*: HMMs libraries: TIGRFAM, Pfam, PRK HMMs). Chr., chromosome. GCF_013415115.1, *V. zhaodongensis* reference genome assembly

The physiological and biochemical characteristics of strain BS253 are detailed in Table 2. Based on conventional phenotypic assays, the isolate was oxidase-positive and catalase-negative. BS253 was classified as moderately halophilic, with optimal growth at 3–5% NaCl, whereas only limited growth was observed at 0%, 15%, and 20% NaCl (Additional file: Fig. S1). Growth occurred between 10 and 40 °C, with an optimum between 20 and 30 °C, and over a pH range of 7–11, with an optimum at pH 8. The strain showed no hydrolytic activity toward urea, starch, or Tween 80.

**Table 2:** Characteristics that differentiate strain BS253 from closely related strains of the*Vreelandella species*

Characteristic	1	2	3	4	5
Site sampling	Tropical coastal water	Montefredane salt pool	Saline Lake	Saline–alkaline soil	Hypersaline–alkaline lake
Cell morphology	Rods 1.5–2.0 μm × 0.2–0.5 μm	Rods 2.3–2.6 μm × 0.6–0.7 μm	Rods 1.3–1.8 μm × 1.3–3 μm	Rods 0.8–1.3 μm × 2.0–3.4 μm	Rods 1.2–2.0 μm × 0.5–0.8 μm
Colony color	Yellow	Beige	Cream	Beige-yellow	Beige/Cream
Motility	+	+	+	+	–
Catalase	+	w+	+	+	–
Temperature range (°C)	4–45^a^	5–50^a^	4–45^b^	4–60^c^	10–40
Optimum temperature (°C)	28^a^	37^a^	30–35^b^	35^c^	20–30
pH range	5–10^a^	7.5–10^a^	6–11^b^	6–12^c^	7–11
Optimum pH	7–7.8^a^	9^c^	7–8^b^	9^c^	8
NaCl range (%)	0–20^a^	0–20^a^	0.5–20^b^	0–15^c^	0–20*
Optimum NaCl	0.5–7^a^	10^a^	1–3^b^	3^c^	3–5
Facultatively anaerobic	–	–	–	–	+
H_2_S production	+	w+	–	+	–
Hydrolysis of Starch	–	–	+	–	–
Urease	+	–	+	–	–
DNA G + C content (mol%)	52.3^b^	52.9^a^	52.5^b^	53.8^c^	53.35
Acid production from:					
D-Glucose	–	+	ND	+	+
Lactose	–	–	+	–	–
Sucrose	–	+	+	+	+
L-Arabinose	–	–	+	–	+
Maltose	–	+	+	–	+
L-Rhamnose	–	w+	–	–	+
D-Mannitol	+	–	–	–	+
Sorbitol	+	–	+	–	+
Inositol	ND	ND	+	–	+
Carbon utilization:					
D-Glucose	–	+	+	+	–
L-Arabinose	–	w+	+	–	–
D-Mannose	+	+	ND	+	–
D-Mannitol	+	ND	–	+	–
N-acetyl-glucosamine	+	ND	ND	ND	*+
Maltose	–	+	+	+	*+
Sodium citrate	+	+	–	+	*+

Strain: 1, *V. venusta* (GCF_015689495.1) (Baumann et al. 1972; Dobson and Franzmann 1996; Kaye et al. 2004; Martinez-Abarca et al. 2021); 2, *V. alkaliphila* (GCF_033842765.1) (Romano et al. 2006); 3, *V. andesensis* (GCF_003989795.1) (Guzmán et al. 2010); 4, *V. zhaodongensis* (GCF_013415115.1) (Jiang et al. 2013); 5, *V. zhaodongensis* BS253 (this study). All data for BS253 strain were generated in this study. +, positive; -, negative; w+, weakly positive, *+ 96 h positive; ND, not determined. *Only limited growth was observed at 0%, 15%, and 20% NaCl

Biochemical profiling using the API 20E and API 20NE systems showed that strain BS253 reduced nitrate and hydrolyzed esculin but tested negative for hydrogen sulfide (H_2_S) production, indole production, Voges–Proskauer reaction, ONPG, gelatin hydrolysis, tryptophan deaminase, arginine dihydrolase, phenylalanine deaminase, lysine decarboxylase, and ornithine decarboxylase. Acid production was detected from D-glucose, sucrose, L-arabinose, maltose, L-rhamnose, D-mannitol, sorbitol, and inositol, whereas lactose was not fermented. Furthermore, N-acetyl-glucosamine, maltose, and citrate were utilized as sole carbon and energy sources.

**Table 3:** Hydrocarbon and plastic degradation genes identified in the *Vreelandella zhaodongensis* BS253 genome

HADEG group	Gene	Mechanism	Compound	Pathway
4_A_Alkanes	AeAB_ahpC	Ae_Aerobic	A_Alkanes	A_Finnerty_pathway
4_A_Alkanes	AeAB_ahpF	Ae_Aerobic	A_Alkanes	A_Finnerty_pathway
4_A_Alkanes	AeAC_blc	Ae_Aerobic	A_Alkanes	A_Hydrocarbon_uptake
4_C_Aromatics	AeCKII_pcaF	Ae_Aerobic	C_Aromatics	C_Catechol_degradation
4_C_Aromatics	AeCKII_pcaJ	Ae_Aerobic	C_Aromatics	C_Catechol_degradation
4_C_Aromatics	AeCRI_nagL	Ae_Aerobic	C_Aromatics	C_Gentisate_degradation
4_C_Aromatics	AeCS_hmgA	Ae_Aerobic	C_Aromatics	C_Homogentisate_degradation
4_C_Aromatics	AeCUIII_nahF	Ae_Aerobic	C_Aromatics	C_Naphthalene_degradation
4_C_Aromatics	AeCY_paaA	Ae_Aerobic	C_Aromatics	C_Phenylacetate_degradation
4_C_Aromatics	AeCY_paaB	Ae_Aerobic	C_Aromatics	C_Phenylacetate_degradation
4_C_Aromatics	AeCY_paaC	Ae_Aerobic	C_Aromatics	C_Phenylacetate_degradation
4_C_Aromatics	AeCY_paaD	Ae_Aerobic	C_Aromatics	C_Phenylacetate_degradation
4_C_Aromatics	AeCY_paaK	Ae_Aerobic	C_Aromatics	C_Phenylacetate_degradation
4_E_Plastics	AeEGA_est	Ae_Aerobic	E_Plastics	E_PBAT
4_E_Plastics	AeEMA_est	Ae_Aerobic	E_Plastics	E_PET

Genes were annotated against the HADEG (Hydrocarbon Aerobic Degradation Enzymes and Genes) database. Each entry lists the HADEG functional group, the gene name, the associated degradation mechanism (Ae, aerobic), the target compound class (A, alkanes; C, aromatics; E, plastics), and the specific metabolic pathway or subpathway to which the gene is assigned. Genes span alkane degradation (Finnerty pathway and hydrocarbon uptake), aromatic compound degradation (catechol, gentisate, homogentisate, naphthalene, phenylacetate, protocatechuate, and toluene pathways), and putative plastic degradation (PBAT and PET)

Regarding antibiotic susceptibility, strain BS253 was resistant to ampicillin, exhibiting bacterial growth after 72 h of incubation at both 50 and 100 µg/mL. In contrast, no bacterial growth was observed in the presence of kanamycin, chloramphenicol, or tetracycline at any of the concentrations or time points evaluated, indicating high susceptibility to these antibiotics under the tested conditions.

### BS253 complete genome properties

**Fig. 2:**
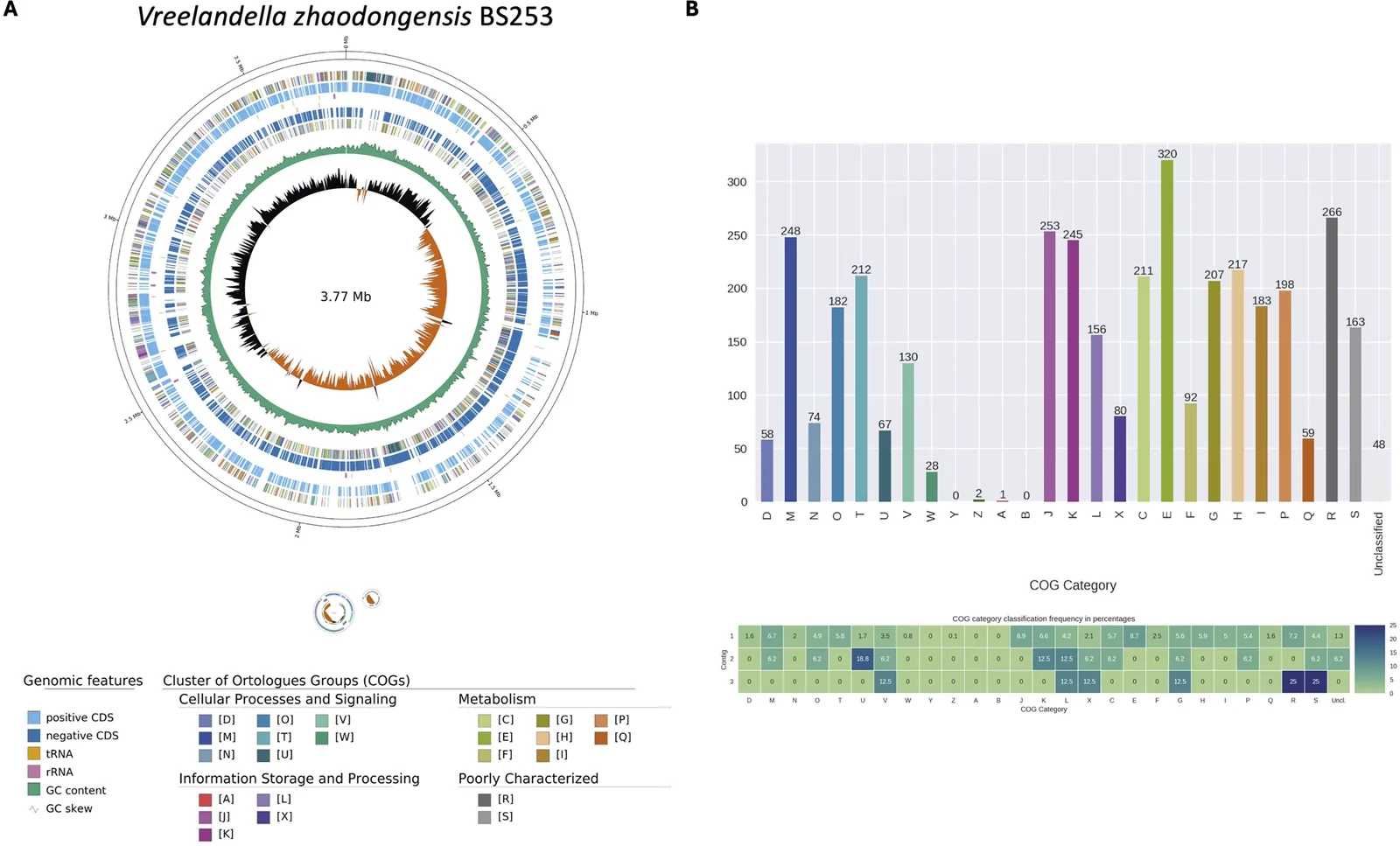
Circular representation of the complete de novo assembled genome of *Vreelandella zhaodongensis* BS253. **A** Overview of the genome of isolate BS253 showing COG functional categories displayed for the metabolism coding region predicted in the assembled genome. **B** A histogram displaying the COG classification categories of every CDS named. *COG* clusters of orthologous groups. Generated by GenoVi (https://github.com/robotoD/GenoVi)

The BS253 genome was sequenced by Illumina and Nanopore sequencing technologies. Using a hybrid assembly approach, we produced a final consensus *de-novo* genome assembly with 3,765,793 bp (Table 1). This complete genome was distributed in a long circular chromosome (Fig. 2a) with G + C content, L90, and N’s bases equal 53.35%, 1, and 0, respectively (Table 1) and approximately 529-fold (Illumina) and 267-fold (Nanopore) coverage. The BS253 genome also showed one copy of each plasmid, one with 28,749 bp and the other with 6,228 bp (Table 1). Coverage-based copy-number estimates indicated a single copy of each plasmid (1.09 and 0.94 for plasmids 1 and 2, respectively; Table 1), consistent with the chromosomal coverage (Tables 1, 2).

The BS253 genome encodes 3,417 genes (3,335 CDS and 3,288 coding) and 60 tRNAs; the rRNA segments comprise six copies of each 5 S, 16 S, and 23 S. The genome showed 4830 GO terms related to molecular function (MF, 2632), biological process (BP, 1606), and cellular component (CC, 592) (Additional file: Fig. S2a-c). Then, the completeness of the *de novo* assembled genome and annotated gene set was assessed with BUSCO to assess using the *halomonas*_*odb12* (Creation date: 2024-11-14, number of genomes: 102, number of BUSCOs: 1250). Among the conserved orthologs, 98.7% were retrieved in both the *de novo* assembled genome and annotated gene set (Complete:98.7% [Single copy: 98.2%, Duplicated:0.6%], Fragmented: 0.4%, Missing: 0.9%, number of BUSCOs: 1250; Additional file: Fig. S3). CheckM analysis confirmed a high-quality assembly, with 99.47% completeness and 0.46% contamination, in agreement with the BUSCO results.

The Clusters of Orthologous Groups of proteins (COGs) for each CDS showed high frequency for amino acid transport and metabolism (E), general function (R), translation, ribosomal structure, and biogenesis (J), cell wall/membrane/envelope biogenesis (M), and transcription (K) (Table S4; Fig. 2b). Both plasmids encode two functional classes: large repeat-containing surface proteins and mobile genetic elements. Plasmid pVBS253_1 (28,749 bp) encodes 14 CDSs, including five large hemagglutinin repeat-containing proteins that share conserved N-terminal repeats but diverge at their C-termini, each followed by a small ORF such as an *SMI1/KNR4* family protein (ACR0PX_RS17140). Four mobile elements are interspersed among these loci, two *Rpn* family recombination-promoting nucleases, an *IS1380* transposase, and an *IS3* transposase expressed through a programmed ribosomal frameshift. Plasmid pVBS253_2 (6,228 bp) encodes eight CDSs, with three predicted proteins nearly identical to internal segments of the pVBS253_1 proteins but divergent at their C-termini. No replication initiator (*rep*), partitioning (*par*), relaxase, type IV secretion, resistance, or biosynthetic genes were annotated on either replicon.

Distinct classes of antibiotic resistance genes are present in the BS253 genome. Small multidrug resistance (SMR), antibiotic efflux pump (*qacG*), vanH gene in vanO cluster (*vanH*), and resistance-nodulation-cell division (RND) antibiotic efflux pump (*rsmA*) were annotated. The resistance mechanisms of these classes are linked to the mechanism of antibiotic efflux and antibiotic target alteration, and drugs like disinfecting agents and antiseptics, glycopeptide antibiotics, fluoroquinolone antibiotics, diaminopyrimidine antibiotics, and phenicol antibiotics (Table S5).

### Phylogenetic analysis and evolutionary history of the strain BS253

**Fig. 3:**
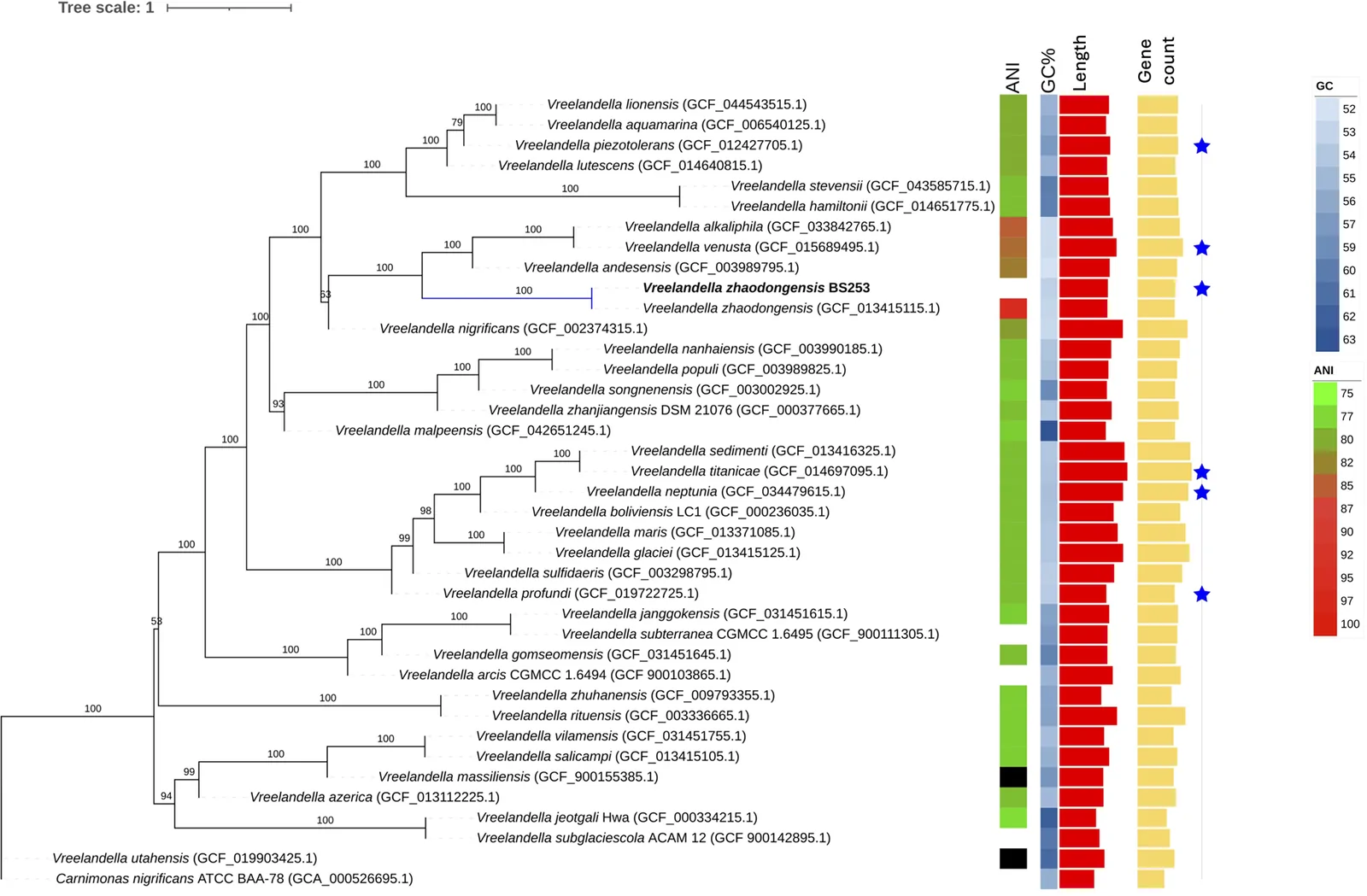
The phylogenetic species tree and the correct placement of the isolate BS253 in the *Vreelandella*. Gene trees were inferred using *RaxML* (ML + LG matrix-based model) for 400 genes in *Phylophlan db*. The final trees were trimmed using *TreeShrink* v1.3.9. The evolutionary species tree was generated using *ASTRAL*. The bootstrap value is shown next to the branches. The *Carnimonas nigrificans* genome was used as an outgroup. ANI values of 253 against reference genomes are shown in the heatmap. Genome statistics are displayed, from left to right: Total length (bp), GC% content, and Gene count. Genomes that are classified as complete sequences are marked with blue stars

We used a multi-marker approach to determine whether the strain BS253 was related to the previously described species in the genus *Vreelandella*, as indicated by *PGAP* after annotation (-*-taxcheck*). The BS253 *de novo* assembled genome best matched *Vreelandella zhaodongensis* (taxid = 1176240) with high confidence. Average nucleotide identity (ANI) analysis between BS253 and *Vreelandella* reference genomes confirmed this classification, with an ANI value of 96.83% relative to *V. zhaodongensis* (GCF_013415115.1). Digital DNA–DNA hybridization (dDDH) analysis further supported BS253 as a strain of *V. zhaodongensis*, yielding a value of 76.4%. 16 S rRNA comparison revealed *V. alkaliphila*,* V. venusta*, and *H*,* hydrothermalis* as the most similar species to BS253 (Table S3). Phylogenetic reconstruction using 400 universal marker genes from PhyloPhlAn placed the strain BS253 within the *V. zhaodongensis* subclade and closely related species such as *V. alkaliphila*,* V. venusta*,* and V. andesensis* with strong bootstrap support (Fig. 3). Our analysis also showed strong support for other clades within the *Vreelandella* genus, further supporting our phylogenetic reconstruction for the group. Genome features, including GC content, genome length, and gene count, were consistent with those reported for other members of the genus *Vreelandella*.

### Secondary metabolites and special metabolism

**Fig. 4:**
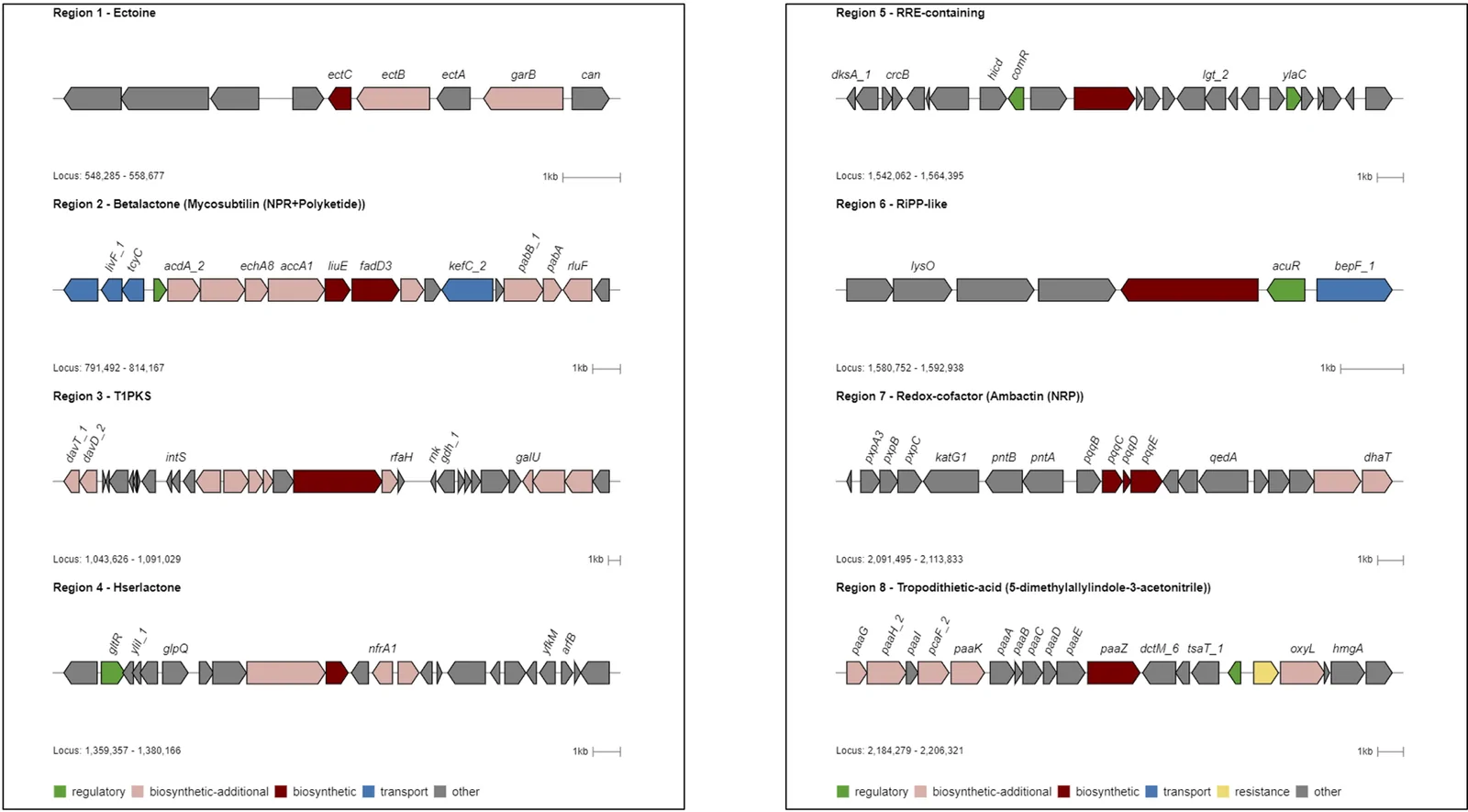
Biosynthetic gene cluster type detected in the BS253 genome. Genetic architecture of eight putative secondary metabolite clusters predicted via antiSMASH analysis. The identified regions encompass pathways for: Region 1, ectoine; Region 2, β-lactone; Region 3, type I polyketide synthase (T1PKS); Region 4, homoserine lactone; Region 5, RRE-containing peptides; Region 6, RiPP-like peptides; Region 7, redox-cofactor; and Region 8, tropodithietic acid. Genes are depicted as arrows indicating the direction of transcription and are color-coded according to their predicted functional roles: core biosynthetic genes (dark red), additional biosynthetic genes (light pink), transport-related genes (blue), regulatory genes (green), resistance genes (yellow), and genes with other or unassigned functions (grey). Genomic locus coordinates are provided below each respective cluster. Scale bar = 1 kb

Genome mining using antiSMASH identified eight putative biosynthetic gene clusters (BGCs) in the genome of strain BS253 (Fig. 4, Tables S6 and S7). These clusters were predicted to encode pathways for diverse classes of specialized metabolites, including ectoine, β-lactone, type I polyketide synthase (T1PKS), homoserine lactone, RRE-containing RiPPs, RiPP-like peptides, redox-cofactor pathways, and tropodithietic acid. The ectoine cluster showed the highest similarity to known clusters, displaying 75% similarity to the ectoine biosynthetic pathway, which is typically associated with osmoprotection in halophilic and halotolerant microorganisms, consistent with the hypersaline origin of strain BS253 (Table S8). A β-lactone cluster 2) exhibited low similarity (20%) to the mycosubtilin biosynthetic cluster, suggesting the potential production of a structurally related but divergent metabolite. Additionally, a large type I polyketide synthase (T1PKS) cluster was detected, containing genes encoding polyketide synthase modules and associated tailoring enzymes, indicating the capacity for the biosynthesis of complex polyketide compounds.

Other predicted clusters included (i) a homoserine lactone-related region potentially involved in quorum–sensing–associated metabolites, an RRE-containing cluster; (ii) a RiPP-like cluster, suggesting the production of ribosomally synthesized and post-translationally modified peptides; and (iii) a redox-cofactor cluster associated with pyrroloquinoline quinone (PQQ) biosynthesis, a cofactor involved in bacterial redox metabolism. Finally, A BGC showed 55% similarity to the tropodithietic acid biosynthetic pathway, a sulfur-containing antimicrobial compound commonly associated with marine bacteria, suggesting a possible role in microbial competition or ecological fitness.

**Fig. 5:**
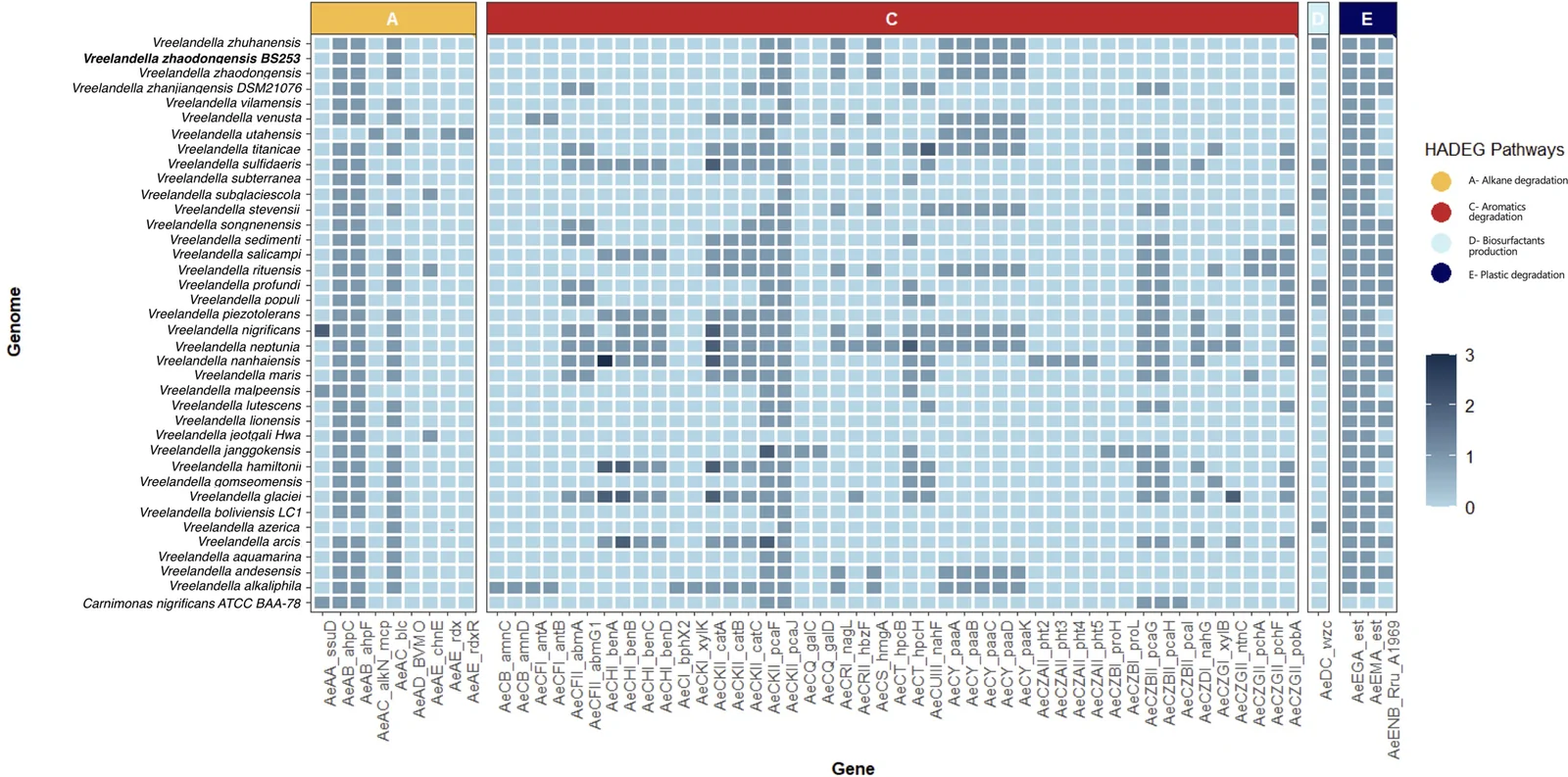
Pathway groups related to alkanes, aromatics, and plastic degradation. Heatmap with the validated genes and the number of hits identified in isolate BS253 and within *Vreelandella*. Gene groups are divided by degradation pathways (A = Alkanes degradation; C = Aromatics degradation; D = Microbial surfactants production; E = Plastics degradation). Isolate BS253 and *V. zhaodongensis* (GCF_013415115.1) are highlighted

As *V. zhaodongensis* BS253 was isolated from a hypersaline lake, we explored genes and metabolic pathways that may contribute to survival under such harsh environmental conditions. HADEG analysis revealed the presence of pathways associated with the degradation of (i) alkanes, (ii) aromatics and (iii) plastic degradation. The heatmap comparison across *Vreelandella* genomes showed that these metabolic pathways are broadly distributed within the genus, although with varying gene abundances. Notably, genes associated with aromatic compound degradation were among the most represented modules, suggesting a conserved metabolic potential for processing complex organic substrates in this group. In the strain BS253, several genes related to the Finnerty pathway (*ahp*C and *ahpF*), hydrocarbon uptake (*blc*), catechol degradation (*pcaF* and *pcaJ*), gentisate degradation (*nagL*), homogentisate degradation (*hmgA*), naphthalene degradation (*nahF*), phenylacetate degradation (*paaA*,* paaB*,* paaC*,* paaD*, and *paaK*), protocatechuate degradation (*pcaG* and *pcaH*), and toluene degradation (*pobA*) were identified. Additionally, genes potentially involved in PBAT/PET plastic degradation (*est*) were detected (Table 3; Fig. 5).

The genome of *V. zhaodongensis* strain BS253 revealed the presence of multiple toxin–antitoxin (TA) loci. A total of 23 putative type II TA pairs were identified across the genome (Additional file: Fig. S4). These systems consist of closely linked genes encoding a stable toxin and its cognate antitoxin protein. The predicted TA modules belong to several well-characterized families (Table S9), including *RelE/ParE*, *VapBC*, *Phd/YefM*, *HigBA*, *HicAB*, and *BrnTA*. Among them, *RelE/ParE*-like systems were the most prevalent, with several toxin genes encoding ribosome-dependent mRNA interferases paired with transcriptional regulator–type antitoxins. Other notable systems include the *VapBC* module, composed of the *VapC* toxin and *VapB* antitoxin associated with plasmid stabilization, and the *Phd*/*YefM*–*RelE* modules, which are commonly linked to stress response and persistence mechanisms in bacteria.

**Fig. 6:**
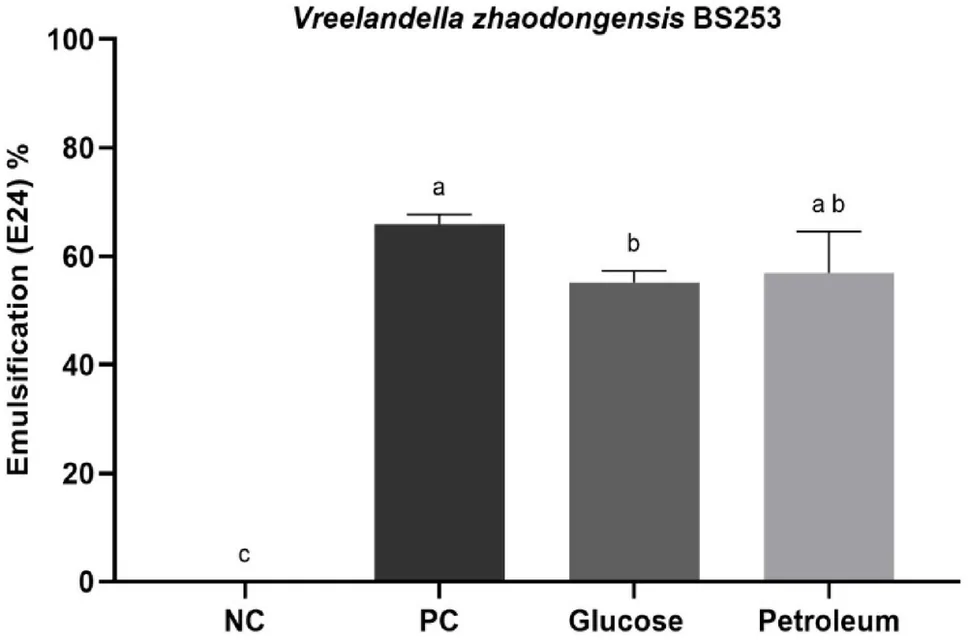
Emulsification index of *Vreelandella zhaodongensis* strain BS253 cultured in MSM medium supplemented with 2% glucose and petroleum. The assay was performed using whole-cell cultures after 5 days of incubation. *NC* negative control (uninoculated culture medium); *PC* positive control (0.5% SDS). Different letters indicate statistically significant differences (*p* < 0.05) according to one-way ANOVA followed by Tukey’s post hoc test

To investigate whether these metabolic features could be associated with microbial surfactant-related activity, we evaluated the ability of strain BS253 to produce emulsifying compounds. Strain BS253 showed positive emulsifying activity when cultivated in MSM medium supplemented with distinct carbon sources. In the emulsification assay, the strain reached an E24 of 55.1% in medium supplemented with 2% glucose and 56.9% when cultivated with petroleum as substrate (Fig. 6). No significant difference was observed between the petroleum treatment and the positive control. Despite the positive results in the emulsification assay, the drop-collapse and oil-spreading assays yielded negative results.

Overall, these results indicate that strain BS253 harbors a diverse repertoire of biosynthetic pathways, highlighting its potential to produce osmoprotectants, signaling molecules, and bioactive secondary metabolites. Type II TA modules are widely distributed in bacterial genomes and are commonly associated with genome and plasmid maintenance, stress responses, and persistence. Their presence in BS253 is consistent with this general distribution; establishing any specific contribution to halotolerance would require comparative or functional analyses beyond the scope of this study.

### Comparative functional genomics with the closest relatives

To determine which features of BS253 are shared with, and which are distinctive of, its closest relatives, the genome was compared with those of *V. zhaodongensis* 9-2T, *V. alkaliphila*, *V. venusta* and *V. andesensis.* Orthologue clustering of the five proteomes defined a pangenome of 4,835 gene families, of which 2,385 were core, 1,257 accessory and 1,193 restricted to a single genome. Strain BS253 contributed 2,426 genes to the core, 678 to the accessory fraction, and 136 strain-specific genes, i.e., 4.2% of its 3,240 distinct proteins (Additional file: Fig. S5A). This is the second smallest strain-specific fraction of the set, close to that of *V. zhaodongensis* 9-2T (131) and well below those of *V. alkaliphila* (244), *V. andesensis* (328) and *V. venusta* (411), indicating that BS253 does not carry an unusually large accessory genome.

COG functional profiles were essentially superimposable across the five genomes (Additional file: Fig. S5B). Amino-acid transport and metabolism (E), translation (J) and cell wall/membrane biogenesis (M) were the most abundant categories in every genome. The largest deviations of BS253 from the mean of the four relatives were modest: an enrichment in defense mechanisms (V; 3.6% versus 2.7%), coenzyme transport and metabolism (H; 6.5% versus 5.9%) and translation (J; 8.3% versus 7.8%), and a depletion in carbohydrate transport and metabolism (G; 5.2% versus 6.2%) and transcription (K; 6.2% versus 7.0%). The overall functional repertoire of BS253 is therefore typical of the genus, and its distinctiveness resides in its accessory, not its core, gene content.

Consistent with this, mobile and defense functions dominated the 136 BS253-specific proteins: 81 had no COG assignment and were mostly annotated as hypothetical or DUF-containing proteins, while among the 55 classified proteins defense mechanisms (V, *n* = 15) and mobilome (X, *n* = 9) together accounted for 44% (Additional file: Fig. S5C). Six of these proteins are plasmid-borne, and on the chromosome the strain-specific genes are not dispersed but clustered into 35 groups, eleven of which contain five or more genes (Additional file: Table S12). The largest is a 39.0 kb island (positions 1,822,581–1,861,557) carrying a complete integrative and conjugative element (ICE): traG, a conjugative transfer ATPase, the TIGR03745, TIGR03749, TIGR03750, TIGR03751, TIGR03752, TIGR03756, TIGR03757 and TIGR03758 ICE protein families, a RAQPRD-family protein, STY4534- and STY4528-family replication proteins and a ParB partitioning protein, together with IS200/IS605 transposases and five type II toxin–antitoxin modules. This element accounts for part of the high number of toxin–antitoxin loci described above and represents the main lineage-specific acquisition of BS253 relative to the other *V. zhaodongensis* genome.

**Fig. 7:**
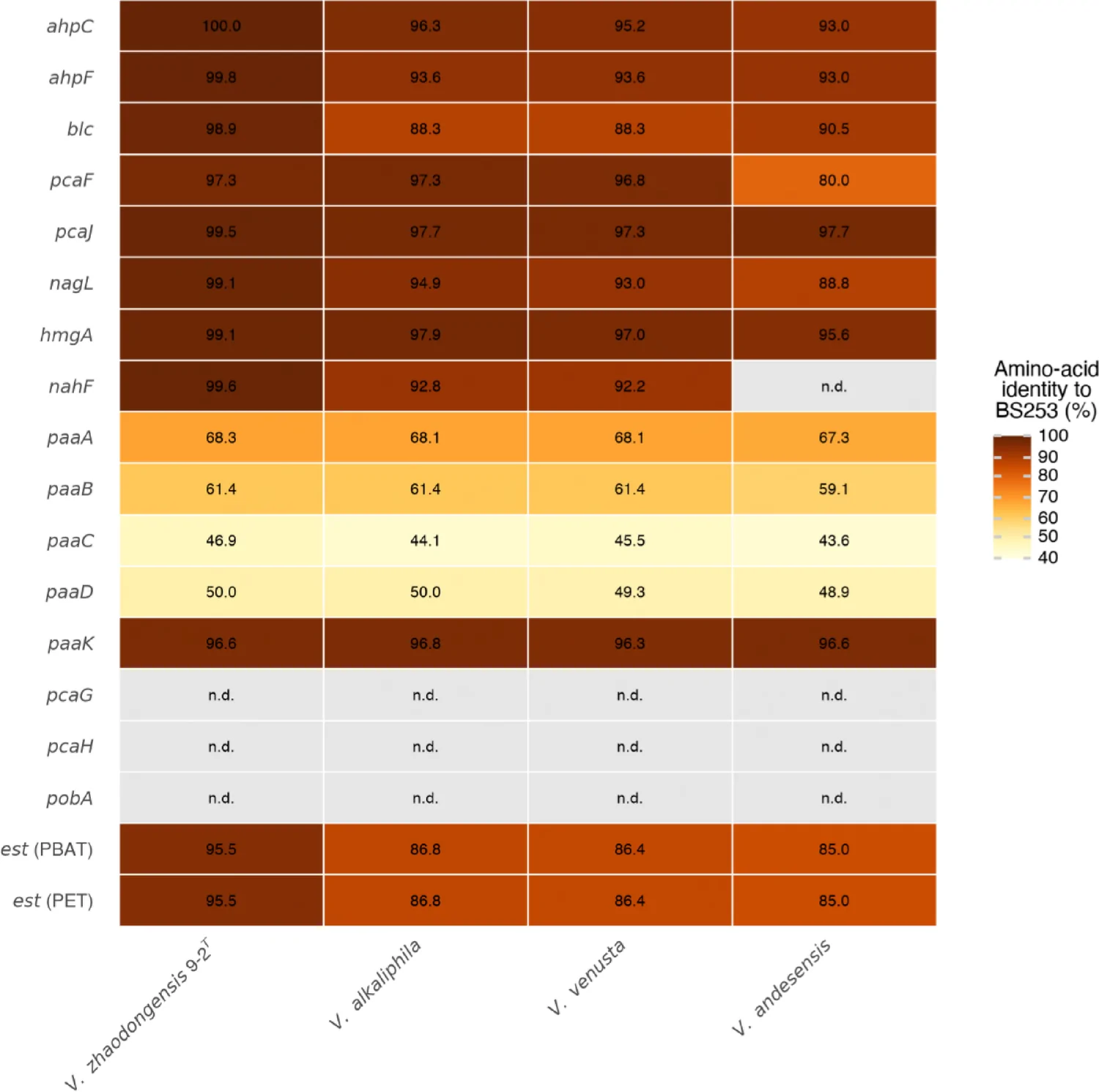
Conservation of the hydrocarbon-, aromatic-, and plastic-degradation genes of strain BS253 in the four closest reference genomes. Cell values are the amino-acid identity (%) between each BS253 protein listed in Table 3 and its reciprocal best BLASTp hit in the indicated genome (e-value ≤ 1 × 10^− 5^; ≥ 50% mutual coverage); query coverage and protein accessions are given in Additional file: Table S10. n.d., no reciprocal best hit detected. *pcaG*, *pcaH* and *pobA* have no orthologue in BS253 or in the four reference genomes: *pcaG* and *pcaH* gave no BLASTp or tBLASTn hit at all, and *pobA* only a 116-residue partial match at 31% identity covering 29% of the reference protein, which does not support orthology. Genes are ordered by degradation pathway as in Table 3

The degradation-related genes listed in Table 3 were then examined individually (Fig. 7; Additional file: Table S10). Fifteen of the eighteen genes were recovered in the BS253 proteome, and all fifteen have reciprocal best-hit orthologues in *V. zhaodongensis* 9-2T. Eleven of them are essentially identical to their 9-2T counterparts (95.5–100% amino-acid identity, median 99.1%, full-length coverage) and share 80.0–97.9% identity with *V. alkaliphila*, *V. venusta* and *V. andesensis*, the only missing orthologue being *nahF* in *V. andesensis*. The remaining four genes, the phenylacetate epoxidase subunits *paaA*, *paaB*, *paaC* and *paaD*, are markedly divergent: 46.9–68.3% identity to 9-2T and 43.6–68.3% across all four reference genomes, whereas the adjacent phenylacetate-CoA ligase *paaK* is 96.3–96.8% identical. The uniform divergence of *paaABCD* across all four relatives, against a background of near identity elsewhere, is consistent with the acquisition of a divergent *paaABCD* variant by BS253. These results show that BS253’s hydrocarbon-, aromatic-, and plastic-degradation potential is a conserved property of this clade rather than a strain-specific trait, and that the genes distinguishing BS253 from its relatives are predominantly associated with mobile genetic elements and anti-phage defense.

## Discussion

Genome sequencing of environmental microorganisms enables the identification of unique biological traits and facilitates the discovery of new biotechnological applications (Katz and Baltz 2016). In this study, we characterized the genome of *V. zhaodongensis* isolated from a hypersaline alkaline lake in Brazil’s Pantanal using hybrid sequencing technologies. In addition, we investigated its genetic content and biosynthetic potential.

The genus *Vreelandella* belongs to the family *Halomonadaceae*. This genus was proposed by de La Haba et al. in 2023 from a taxonomic re-evaluation of the phylogenetically heterogeneous *Halomonas* (de la Haba et al. 2023). Using a multi-marker phylogenetic approach and genome-relatedness indices, the *de novo* assembled genome of strain BS253 showed the highest similarity to *V. zhaodongensis*, confirming the phylogenetic structure proposed by de la Haba et al. in 2023. BS253 is confirmed as a strain of *V. zhaodongensis*, with ANI of 96.83% (above 95–96% species boundary) and dDDH 76.4% (Meier-Kolthoff et al. 2013, 2014). Currently, there are only two deposited genome sequences for this species on the NCBI genome database: from the study that described *V. zhaodongensis* (reference genome, ASM1341511v1) (Jiang et al. 2013), and another deposited by the Global Catalogue of Microorganisms (GCM) 10 K type strain sequencing project (ASM4266499v1) (Wu and Ma 2019). Both genomes are assembled at a contig level, with a total of 25 scaffolds. In addition, for the 201 deposited genomes of the *Vreelandella* genus present at the NCBI genome database, only 52 displayed chromosome-level scaffolds, reinforcing the need for complete genome assemblies for proper functional and phylogenetic analysis of this genus. The genome of BS253 was sequenced and assembled by combining high-coverage Nanopore reads and high-coverage Illumina reads from the same genome using the steps in the *Trycycler* pipeline. We assembled a gap-free, highly accurate, complete genome with a 3,765,793 bp circular chromosome representing the first complete DNA sequence for *V. zhaodongensis*, providing the research community with additional information for this distinct halophilic bacterial group.

Beyond its genomic characteristics, the ecological origin of BS253 provides insights into its physiological adaptations. Close phylogenetic relatives of strain BS253 have been recovered from tropical coastal water, salt pools, saline lakes, and saline-alkaline soils. The Pantanal region harbors numerous hypersaline and alkaline lagoons that act as reservoirs of extremophilic microbial diversity, selecting for organisms adapted to extreme salinity, pH, and temperature fluctuations that can be exploited for biotechnological applications (Guerreiro et al. 2019). Consistent with its environmental origin, strain BS253 showed a clear preference for saline conditions and tolerated neutral to alkaline pH. Growth was substantially higher at intermediate NaCl concentrations, with an optimum at 3–5%, although limited growth was also observed at 0%, 15%, and 20% NaCl. Therefore, BS253 does not exhibit an absolute requirement for NaCl under the tested conditions. Nevertheless, its optimal growth in the presence of salt supports its classification as moderately halophilic. The strain also showed optimal growth at pH 8 and between 20 and 30 °C. Together, these traits indicate that BS253 is physiologically adapted to saline-alkaline environments, while also showing a narrower temperature preference than some closely related *Vreelandella* strains.

When compared with phylogenetically related reference strains, BS253 showed a distinct phenotypic profile, including lack of motility, absence of catalase activity, and facultative anaerobic growth. Motility, catalase reaction, and similar physiological characters are known to vary among strains within the *Halomonadaceae* and are not regarded as definitive taxonomic markers (Mata et al. 2002; Arahal et al. 2007). Both aerobic and facultatively anaerobic metabolism have been reported within the genus *Vreelandella* (de la Haba et al. 2023), supporting the phenotypic variability observed among related strains. The large repeat proteins encoded on both plasmids belong to the TpsA/filamentous haemagglutinin family, members of which mediate functions ranging from adherence to interbacterial intoxication (Guérin et al. 2017; Nash and Cotter 2019). Their conserved-N-terminal/variable-C-terminal organization, together with the small ORFs adjacent to each repeat protein and the truncated homologues carried by pVBS253_2, is compatible with polymorphic toxin cassettes and their cognate immunity partners (Zhang et al. 2012; Ruhe et al. 2020). These features may reflect adaptations to specific microhabitats within hypersaline alkaline sediments, where oxygen availability, salinity, and nutrient composition can vary.

The biochemical profiles (API 20E and 20NE) further supported the metabolic versatility of strain BS253. The ability to produce acids from several carbohydrates, including D-glucose, sucrose, L-arabinose, maltose, L-rhamnose, D-mannitol, sorbitol, and inositol, together with the utilization of N-acetyl-glucosamine, maltose, and citrate as sole carbon and energy sources, indicates that BS253 can metabolize diverse organic substrates. This phenotypic profile is consistent with the genome annotation, which revealed a substantial number of genes assigned to carbohydrate transport and metabolism, amino acid transport and metabolism, energy production and conversion, and membrane/envelope-related functions. Such metabolic flexibility may contribute to the survival of BS253 in hypersaline alkaline lakes, where nutrient availability, salinity, pH, and oxygen conditions can fluctuate seasonally. This metabolic versatility extends to the degradation of complex hydrophobic substrates, a process often assisted by microbial surfactants that improve substrate bioavailability and are relevant to bioremediation and enhanced oil recovery (Corti Monzón et al. 2018). In our study, genome analysis of BS253 revealed pathways related to alkane, aromatic, and plastic degradation, consistent with its capacity to process complex organic substrates. The strain exhibited emulsifying activity when cultivated in MSM supplemented with either glucose (E24 = 55.1%) or petroleum (E24 = 56.9%), with no significant difference between the petroleum treatment and the positive control. However, the negative drop-collapse and oil-spreading results indicate that the phenotype is more consistent with the production of emulsifying compounds than with a low-molecular-weight biosurfactant. In addition, no canonical genes or biosynthetic clusters of well-characterized biosurfactants were detected in the genome, suggesting the presence of non-classical or still poorly characterized emulsifying molecules.

The interpretation of BS253 as an emulsifier-producing strain is further supported by a recent physiological and transcriptomic study of the same isolate, in which inorganic carbon availability modulated emulsification activity and altered the expression of genes associated with membrane transport, envelope-related functions, and extracellular polymer-associated pathways (Napp et al. 2026a). Importantly, that study also reported that the extracellular emulsification-active material retained activity across broad ranges of salinity, temperature, pH, and pressure, reinforcing the potential relevance of BS253-derived extracellular compounds for saline-alkaline bioprocesses. However, neither the present study nor the previous transcriptomic analysis demonstrates direct autotrophic CO_2_ fixation or establishes the chemical identity and molecular composition of the emulsification-active material, which will require targeted purification and structural characterization.

## Conclusion

The isolation of microbial organisms from extreme environments is pivotal for the identification of new strains with potential for ecological and biotechnological applications (Finore et al. 2015; Shu and Huang 2022). Through integrated genomic, phylogenomic, and phenotypic analyses, BS253 was characterized as a strain of *V. zhaodongensis*, displaying adaptations to saline-alkaline conditions and genes associated with hydrocarbon and plastic degradation, as well as secondary metabolite biosynthesis. The observed emulsifying activity under defined culture conditions further supports the biotechnological relevance of this strain. Together with previous physiological and transcriptomic evidence showing that BS253 responds to inorganic carbon availability, the complete genome reported here expands the genomic and functional knowledge of the genus *Vreelandella* and provides a foundation for future studies, including targeted assays to evaluate its potential in carbon-associated bioprocesses, industrial applications, and bioremediation.

## Supplementary Information

Below is the link to the electronic supplementary material.

Supplementary Material 1

Supplementary Material 2. Table S1 - Project information. Table S2 - Vreelandella genomes references. Metadata for the genomic data accessed in NCBI. Table S3–16rRNAs hits. Table S4 - COGs categories. Table S5 - CARD genes. List of annotated CARD genes in V. zhaodongensis BS253. Table S6 – Complete list of secondary metabolite biosynthesis gene clusters presented in V. zhaodongensis BS253. Full list of clusters annotated using antiSMASH. See methods for details. Table S7 - Complete list of genes within each secondary metabolite biosynthesis gene cluster annotated in V. zhaodongensis BS253. See methods for details. Table S8 – Ectoine gene cluster nodes. Annotation of V. zhaodongensis BS253 ectoine gene cluster. Table S9 - Toxin–antitoxin system in V. zhaodongensis BS253. List of genes of TA pairs annotated in the genome. Table S10. Conservation of the degradation-related genes of Table 3 in the four closest reference genomes. Amino-acid identity and query coverage of each BS253 protein and its reciprocal best BLASTp hit (e-value ≤ 1 × 10-5; ≥ 50 % mutual coverage). “none”, no reciprocal best hit; “not detected”, the HADEG reference protein has no homologue in the BS253 proteome (BLASTp, e-value ≤ 1 × 10-5) or in the BS253 chromosome (tBLASTn, e-value ≤ 1 × 10-3). Copies in BS253; number of distinct BS253 proteins assigned to that HADEG gene. Table S11. Genes found exclusively in strain BS253 among the five genomes compared. The 136 proteins of BS253 without an orthologue in V. zhaodongensis 9-2T, V. alkaliphila, V. venusta, or V. andesensis (OrthoFinder v3.1.5), with their genomic location, product, and COG assignment. Coordinates refer to the first CDS locus of each protein on the indicated replicon. Table S12. COG functional profile of the five genomes. Number (n) and percentage (%) of COG-classified proteins assigned to each functional category (COGclassifier v2.0.0; RPS-BLAST against the NCBI CDD COG profiles, e-value ≤ 0.01). Percentages are relative to the COG-classified proteins of each genome. Table S13. Chromosomal clusters of BS253-specific genes. Strain-specific genes on the BS253 chromosome grouped with a maximum intergenic distance of 20 kb; clusters of three or more genes are listed. Up to five representative products are shown per cluster; the complete gene list is given in Table S10

## Data Availability

This work is registered in Sistema Nacional de Gestão do Patrimônio Genético e do Conhecimento Tradicional Associado (SisGen) under Project code A5C07E4. The assembled genome is available in the accession GCF_051175405.1.
