# Supplementary material for: Functional genomic analysis of Vreelandella zhaodongensis BS253: a new isolate from hypersaline lakes from Brazilian Pantanal
Source: Arch Microbiol. 2026 Oct 31;209(1):41. doi: 10.1007/s00203-026-05318-2 (PMC13723453; doi:10.1007/s00203-026-05318-2)
Supplement: Supplementary file 1 [file 203_2026_5318_MOESM1_ESM.docx]

**Figure S1. Effect of physicochemical conditions on the growth of strain BS253 after 24 h and 48 h of incubation under different parameters. (A)** NaCl concentrations (%), **(B)** temperature (°C), and **(C)** initial pH values. Bars represent the mean values, and different letters indicate statistically significant differences (*p* < 0.05) according to Tukey's test.

**Figure S2.** **Functional annotation of the complete *de novo* assembled genome of *V. zhaodongensis* BS253. (A-C)** Top 20 most represented GO functional annotations annotated using PGAP. Panels represent GO categories for Molecular Function (MF, Panel A), Biological Process (BP, Panel B), and Cellular Component. Each category is described on the y-axis, while the number of genes in each category is displayed in horizontal bars along the x-axis. The bars are colored according to the percentage of each category (Adj-Frequency, Adj-freq).

**Figure S3. BUSCO evaluation results for the de novo assembled genomic sequence and the annotated gene set.** BUSCO tool v5.8.2 using the specific reference halomonas_odb12 (Creation date: 2024-11-14, number of genomes: 102, number of BUSCOs: 1250). Gene set 253 (C:98.7% [S:98.2%, D:0.6%], F:0.4%, M:0.9%, n:1250) and genome 253 (C:98.7% [S:98.2%, D:0.6%], F:0.4%, M:0.9%, n:1250).

**Figure S4. Toxin-antitoxin pairs in the genome of isolate 253.** The annotated genome showed 23 putative TA pairs annotated in the TADB database. Blue, putative toxin genes; Brown, putative antitoxin genes. Generated using *TAfinder* 2.0 (<https://bioinfo-mml.sjtu.edu.cn/TADB3/TAfinder.php>).

**Figure S5.** Comparative functional genomics of *Vreelandella zhaodongensis* BS253 and its four closest relatives. **(A)** Partition of the protein-coding gene content of each genome into core (orthogroups present in all five genomes), accessory (two to four genomes), and strain-specific (a single genome) families; the number of strain-specific genes is given beside each bar. **(B)** COG functional profile of the five genomes, expressed as the percentage of COG-classified proteins assigned to each functional category; category letters follow the NCBI COG nomenclature and are expanded in panel C and in Table S11. **(C)** COG functional categories of the 136 proteins found exclusively in BS253; defense mechanisms (V) and mobilome (X), highlighted in red, are the two most represented assigned categories. Orthogroups were inferred with OrthoFinder v3.1.5 and COG categories assigned with COGclassifier v2.0.0. Reference genomes: *V. zhaodongensis* 9-2T (GCF_013415115.1), *V. alkaliphila* (GCF_033842765.1), *V. venusta* (GCF_015689495.1), and *V. andesensis* (GCF_003989795.1).
